# Diversity and evolution of ABC proteins in mycorrhiza-forming fungi

**DOI:** 10.1186/s12862-015-0526-7

**Published:** 2015-12-28

**Authors:** Andriy Kovalchuk, Annegret Kohler, Francis Martin, Fred O. Asiegbu

**Affiliations:** Department of Forest Sciences, University of Helsinki, P.O. Box 27, FIN-00014 Helsinki, Finland; UMR 1136, INRA/Université de Lorraine, Interactions Arbres/Microorganismes, INRA, Institut National de la Recherche Agronomique, Centre INRA de Nancy, 54280 Champenoux, France

**Keywords:** ABC transporters, Fungi, Mycorrhiza, Membrane transport, Protein family evolution

## Abstract

**Background:**

Transporter proteins are predicted to have an important role in the mycorrhizal symbiosis, due to the fact that this type of an interaction between plants and fungi requires a continuous nutrient and signalling exchange. ABC transporters are one of the large groups of transporter proteins found both in plants and in fungi. The crucial role of plant ABC transporters in the formation of the mycorrhizal symbiosis has been demonstrated recently. Some of the fungal ABC transporter-encoding genes are also induced during the mycorrhiza formation. However, no experimental evidences of the direct involvement of fungal ABC transporters in this process are available so far. To facilitate the identification of fungal ABC proteins with a potential role in the establishment of the mycorrhizal symbiosis, we have performed an inventory of the ABC protein-encoding genes in the genomes of 25 species of mycorrhiza-forming fungi.

**Results:**

We have identified, manually annotated and curated more than 1300 gene models of putative ABC protein-encoding genes. Out of those, more than 1000 models are predicted to encode functional proteins, whereas about 300 models represent gene fragments or putative pseudogenes. We have also performed the phylogenetic analysis of the identified sequences. The sets of ABC proteins in the mycorrhiza-forming species were compared to the related saprotrophic or plant-pathogenic fungal species. Our results demonstrate the high diversity of ABC genes in the genomes of mycorrhiza-forming fungi. Via comparison of transcriptomics data from different species, we have identified candidate groups of ABC transporters that might have a role in the process of the mycorrhiza formation.

**Conclusions:**

Results of our inventory will facilitate the identification of fungal transporters with a role in the mycorrhiza formation. We also provide the first data on ABC protein-coding genes for the phylum Glomeromycota and for orders Pezizales, Atheliales, Cantharellales and Sebacinales, contributing to the better knowledge of the diversity of this protein family within the fungal kingdom.

**Electronic supplementary material:**

The online version of this article (doi:10.1186/s12862-015-0526-7) contains supplementary material, which is available to authorized users.

## Background

The family of ATP-binding cassette (ABC) proteins encompasses a large and diverse assemblage of proteins that are ubiquitously present in all living organisms [1]. As their name suggests, their principal common feature is the presence of an ATP-binding domain. The domain provides the energy required by ABC proteins to perform their biological functions by hydrolysing ATP. A large fraction of ABC proteins, known as ABC transporters, are implicated in transport of diverse substrates across biological membranes (Table 1) [2–4]. Soluble ABC proteins are not involved in transmembrane transport, but they play an essential role in vital cellular processes, e.g. ribosome biogenesis and mRNA translation (Table 1) [5]. Most of our current knowledge is based on the results of characterization of ABC transporters in two yeast species, baker’s yeast *S. cerevisiae* and fission yeast *Schizosaccharomyces pombe*. Those data were of enormous importance for understanding of the biological significance of ABC transporters, in general. However, both mentioned species are characterised by reduced sets of ABC transporters, lacking many of groups that are otherwise widespread among filamentous fungi. Existing information on function of ABC transporters in filamentous ascomycetes is very scarce and fragmentary, with most of the experiments being designed to assess the role of individual transporters in drug resistance, whereas ABC transporters of basidiomycetes have almost completely escaped the attention of experimental scientists.

**Table 1:** Biological functions of different groups of fungal ABC proteins

Group			Function	References
ABC-A			Biological function in fungi unknown, but *Magnaporthe grisea* transporter Abc4 is required for appresoria formation. Mammalian ABC-A transporters are involved in lipid transport and metabolism	[29–31]
ABC-B	full	ABCB1	Export of mating pheromones	[32–34]
		ABCB2	Multidrug resistance; a specific group of ABCB transporters in ascomycetes (but not in *Agaricomycotina*) is associated with the biosynthesis cluster for the hydroxamate-type siderophores and might have a role in their metabolism.	[14, 35–37]
	half	ABCB3	Export of the mitochondria-synthesized precursors of iron-sulfur (Fe/S) clusters; localised to the inner membrane of mitochondria	[38, 39]
		ABCB4	Heavy-metal resistance; localized to the vacuolar membrane	[40–42]
		ABCB5	Transporter of the inner mitochondrial membrane involved in the export of peptides released upon proteolysis by m-AAA protease	[43, 44]
		ABCB6	Unknown	
ABC-C	ABCC1		Vacuolar import of phosphatidylcholine; bile acid transport	[45, 46]
	ABCC2		Unknown	
	ABCC3		Unknown	
	ABCC4		Unknown	
	ABCC5		Unknown; often associated with the secondary metabolism gene clusters	[14, 15, 36]
	ABCC6		Heavy metal resistance; vacuolar uptake of glutathione conjugates; localized to vacuolar membrane	[42, 47–49]
	ABCC7		Drug resistance; heavy metal resistance	[50–52]
ABC-D	ABCD1		Import of long-chain fatty acids into peroxisomes	[53]
	ABCD2		Import of long-chain fatty acids into peroxisomes	[53]
	ABCD3/4		Unknown	
ABC-E			Ribosome biogenesis and assembly of translational pre-initiation complexes	[54, 55]
ABC-F	ABCF1		Biogenesis of 40S and 60S ribosomal subunits	[56]
	ABCF2		Regulation of mRNA translation; positive regulator of Gcn2p kinase activity	[57]
	ABCF3		Unknown	
	ABCF4		mRNA export factor	[58]
	ABCF5		Translation elongation factor	[59, 60]
ABC-G	ABCG1-5		Pleiotropic drug resistance; weak organic acid tolerace; export of toxic metabolites, sterol uptake, translocation of membrane phospholipids and quorum sensing	[37, 61]
	ABCG6		Unknown	
	ABCG7		Unknown; are characterised by a presence of EGF domain	[14, 15]
ABC-I	ABCI1		Part of the CCR4-NOT transcriptional regulatory complex	[62]
	ABCI2		Unknown	
	ABCI3		Unknown	

Numerous species of fungi are known to form mycorrhizae, symbiotic interactions between fungi and higher plants. It is estimated that more than 90 % of all plant species, including forest trees, wild grasses and many crops are participating in mycorrhizal interactions [6]. Mycorrhiza is a rather broad term that describes structurally heterogeneous types of interactions between fungi and roots of higher plants. In case of ectomycorrhiza, fungal hyphae surround the root tips and can grow between epidermal cells, but never enter cell lumen. On contrary, endomycorrhiza-forming fungi penetrate inside living cells of the root epidermis and cortex [6]. Endomycorrhizae can be further subdivided into ericoid, arbutoid, orchid and arbuscular mycorrhiza. Mycorrhiza-forming fungi are also very diverse taxonomically, as they represent different evolutionary lineages from three fungal phyla, Glomeromycota, Ascomycota and Basidiomycota. Phylogenetic analysis indicates that mycorrhizal symbionts have evolved independently in different lineages of fungi [7]. Establishment of mycorrhizal interaction is a complex process that requires a continuous nutrient and signal exchange between the partners [6]. Transporter proteins are expected to play a key role in this process. Details of those interactions are gradually being elucidated, but many of the involved molecular components remain unknown. A number of works on different plant models have shown that specific plant ABC transporters are required for the formation of arbuscular mycorrhiza, a widespread type of mutualistic interaction between plants and fungi of the phylum Glomeromycota [8–10], and, furthermore, it was demonstrated that the corresponding transporter acts as strigolactone exporter in petunia [10]. It is not known yet whether any of fungal ABC transporters participate in the formation of mycorrhizal interactions.

To provide insight into the diversity of mechanisms for the mycorrhizal symbiosis, the Mycorrhizal Genomic Initiative (MGI) consortium [11] performed the sequencing and analysis of a phylogenetically and ecologically diverse set of mycorrhizal fungal genomes. The set of species sequenced by MGI consortium includes fungi engaged in the various types of mycorrhizal symbiotic interactions (e.g., arbuscular, ericoid, orchid mycorrhizae and ectomycorrhiza). The species selected by MGI consortium belong to the phyla Glomeromycota, Ascomycota and Basidiomycota. They represent three different classes of Ascomycota (Leotiomycotina, Dothideomycotina and Pezizomycotina), and five orders within the class of Agaricomycotina (Basidiomycota) (Table 2). The generated data provide an excellent opportunity for the comparative genomics analysis of evolutionary adaptations of fungi to the symbiosis with plants.

**Table 2:** Distribution of subfamilies of ABC proteins among analysed species of mycorrhiza-forming fungi

Species	Phylum	Class	Order	ABC-A	ABC-B full	ABC-B half	ABC-C	ABC-D	ABC-E	ABC-F	ABC-G	Others	Total
*Rhizophagus irregularis* DAOM 181602	Glomeromycota	Glomeromycotina	Glomerales	0	8(9)^a^	3	9(11)	4(5)	1	3	4(15)	2	34(49)
*Meliniomyces bicolor* E	Ascomycota	Leotiomycotina	Helotiales	3	10(13)	5	12(19)	2	1	5	13	2	53(63)
*Meliniomyces variabilis* F				3	9	5	26(28)	2	1	5	15	2	68(70)
*Oidiodendron majus* Zn				4	8(10)	7	25(26)	2	1	5	18	2	72(75)
*Cenococcum geophilum* 1.58	Ascomycota	Dothideomycotina	*incertae sedis*	1(3)	5(7)	4	8	2	1	5	7	2	35(39)
*Tuber melanosporum* Mel28	Ascomycota	Pezizomycotina	Pezizales	1	5	4	4	2	1	5	8	2	32
*Choiromyces venosus* 120613-1				1	5	4	4	2	1	5	8(11)	3	33(36)
*Terfezia boudieri* S1				1	4	5	5	2	1	4	5(6)	2	29(30)
*Amanita muscaria* Koide	Basidiomycota	Agaricomycotina	Agaricales	1	6(8)	5(7)	9(10)	2(4)	1	3	4(11)	2	33(47)
*Cortinarius glaucopus* AT 2004 276				1	2(9)	8(10)	21(33)	0	1(3)	6(14)	5(9)	3	47(82)
*Hebeloma cylindrosporum* h7				3	3	7	13(14)	2	1	5	7	3	44(45)
*Laccaria amethystina* LaAM-08-1				1(2)	5(17)	7(10)	7(29)	2(4)	1	5(6)	6(13)	3(8)	37(90)
*Laccaria bicolor* S238N-H82				1	5(11)	7	8(14)	2	1	5	8(17)	6(8)	43(66)
*Tricholoma matsutake* 945				1	2(8)	12(13)	10(23)	2	1	5	8(13)	3	44(69)
*Boletus edulis*	Basidiomycota	Agaricomycotina	Boletales	1	2(3)	7	6(13)	2	1	4	4(9)	3(4)	30(44)
*Paxillus involutus* ATCC 200175				1	4(10)	7(8)	8(14)	2	1	5	8(12)	3	39(56)
*Paxillus rubicundus* Ve08.2 h10				1	3(4)	7(8)	6(8)	2	1	5	7(8)	3	35(40)
*Pisolithus microcarpus* 441				0	5(17)	6	7(11)	3	1	3(7)	4(5)	3	32(53)
*Pisolithus tinctorius* Marx 270				0	5(6)	6(8)	16(18)	2	1	3	6	3	42(47)
*Scleroderma citrinum* Foug A				0	6	10(11)	10(13)	2	1	3	3	3	38(42)
*Suillus luteus* UH-Slu-Lm8-n1				1(5)	4(6)	11(13)	7(8)	2(3)	1	5(7)	6(8)	3	40(54)
*Piloderma croceum* F 1598	Basidiomycota	Agaricomycotina	Atheliales	2	7(9)	9	9(16)	2	1	5	10(16)	3	48(63)
*Tulasnella calospora* AL13/4D	Basidiomycota	Agaricomycotina	Cantharellales	1	7	7	9(11)	2	1	5(6)	5(6)	3	40(44)
*Piriformospora indica* DSM 11827	Basidiomycota	Agaricomycotina	Sebacinales	1	3(4)	6	11(13)	2	1	4	4	3	35(38)
*Sebacina vermifera* MAFF 305830				1	6	6	13(14)	2	1	4	3	3	39(40)

^a^numbers in parentheses include putative pseudogenes and partial sequences

**Table 3:** List of ABC-protein encoding genes significantly up-regulated in mycorrhiza-forming mycelium (MYC) as compared to free-living mycelium (FLM)

Species	Protein model	Subfamily	Gene name	Fold change	*p*-value	Mean FLM	Mean MYC
Laccaria bicolor	601729	ABC-B (FL)	LacbiABCB2.1b	6.08	0	1097	6671
	671922	ABC-B (HT)	LacbiABCB6.1	36.94	1.82E-14	957	35334
	327599	Ydr061w	LacbiABCI2.1a	7.89	1.50E-05	328	2590
	298087	Ydr061w	LacbiABCI2.1d	5.90	0	504	2972
Oidiodendron maius	101847	ABC-A	OidmaABCA1.2	3.66	1.35E-05	5.5	20.1
	104750	ABC-B (FL)	OidmaABCB2.3a	3.60	3.02E-32	35.0	125.9
	101864	ABC-C	OidmaABCC3.1	2.51	9.82E-05	14.5	36.5
	106945	ABC-C	OidmaABCC5.1 m	14.29	1.49E-12	5.1	72.5
	173820	ABC-C	OidmaABCC5.1n	8.32	0.029	1.0	8.2
	102203	ABC-C	OidmaABCC7.1a	2.11	0.004	13.7	29.0
	119877	ABC-G	OidmaABCG6.2a	73.40	3.02E-20	0.5	33.9
	123273	ABC-G	OidmaABCG6.2b	15.31	6.58E-20	6.2	95.5
Sebacina vermispora	326231	ABC-B (HT)	SebveABCB6.1a	2.63	5.24E-05	13.2	34.6
	19794	ABC-B (HT)	SebveABCB6.1b	2.42	9.07E-05	11.5	27.9
	127786	ABC-B (HT)	SebveABCB6.3	6.44	8.34E-05	7.2	46.3
	116826	ABC-C	SebveABCC3.2e	2.06	0.002	13.6	28.1
	29919	ABC-C	SebveABCC7.3	3.79	2.17E-13	12.8	48.7
	71362	ABC-F	SebveABCF1.1	2.39	5.06E-06	66.3	158.9
	333124	ABC-F	SebveABCF2.1	2.68	3.60E-13	26.6	71.2
	17618	ABC-G	SebveABCG6.1	2.98	5.32E-06	35.4	105.7
	174708	Ydr061w	SebveABCI2.1	2.87	1.08E-04	10.0	28.6
	69180	Ydr061w	SebveABCI3.1	2.03	4.37E-04	39.1	79.5
Tulasnella calospora	19492	ABC-B (FL)	TulcaABCB2.1a	2.11	2.91E-05	27.7	58.5
	207894	ABC-B (FL)	TulcaABCB2.1d	2.46	1.80E-04	32.4	79.6
	161653	ABC-B (FL)	TulcaABCB2.1e	2.43	0.001	18.4	44.8
	22503	ABC-B (HT)	TulcaABCB6.1a	2.13	0.014	7.4	15.8
	78312	ABC-B (HT)	TulcaABCB6.1b	3.75	6.96E-04	8.0	30.0
	229331	ABC-B (HT)	TulcaABCB6.3b	2.84	0	37.5	106.4
	3597	ABC-C	TulcaABCC6.1	4.39	0.002	20.1	88.1
	22078	ABC-C	TulcaABCC7.2	4.99	1.85E-04	4.4	22.1
	72157	ABC-F	TulcaABCF5.1	58.44	0.021	5.2	302.4
	80372	ABC-G	TulcaABCG4.1b	5.72	5.80E-06	17.4	99.3
	69109	Ydr061w	TulcaABCI2.1	4.67	4.32E-05	4.0	18.6
	243146	Ydr061w	TulcaABCI3.1	3.56	4.77E-09	14.3	50.9
Suillus luteus	17399	ABC-A	SuiluABCA1.1	4.16	4.23E-10	10.5	43.7
	621910	ABC-A	SuiluABCA1.1	3.51	5.79E-05	6.5	22.8
	797578	ABC-B (FL)	SuiluABCB2.1a	2.12	7.14E-08	34.6	73.4
	107869	ABC-F	SuiluABCF3.1	2.28	1.08E-05	19.8	45.2
Paxillus involutus	111294	ABC-B (FL)	PaxinABCB2.1c	4.21	5.22E-05	6.0	25.2
	80655	ABC-G	PaxinABCG2.1e	11.01	0.004	10.2	112.1
	117609	ABC-G	PaxinABCG2.1f	4.68	4.59E-07	7.5	35.0
Piloderma croceum	99185	ABC-B (FL)	PilcrABCB2.1e	3.63	1.06E-46	52.2	189.1
	4675	ABC-B (HT)	PilcrABCB6.2a	3.20	1.61E-06	9.0	28.7
	603979	ABC-G	PilcrABCG6.2	11.34	6.80E-07	1.2	13.3
Hebeloma cylindrosporum	19666	ABC-C	HebcyABCC7.2b	5.61	1.09E-05	3.4	18.9
	449030	ABC-D	HebcyABCD1.1	3.22	0.020	6.2	20.0
	448391	ABC-G	HebcyABCG6.2	5.99	3.75E-04	9.9	59.4

**Table 4:** List of ABC-protein encoding genes significantly down-regulated in mycorrhiza-forming mycelium (MYC) as compared to free-living mycelium (FLM)

Species	Protein model	Subfamily	Gene name	Fold change	*p*-value	Mean FLM	Mean MYC
Laccaria bicolor	633182	ABC-B (FL)	LacbiABCB1.1a	-2.68	0	10110	3774
	617531	ABC-B (FL)	LacbiABCB1.1b	-9.59	6.43E-06	9869	1029
	633953	ABC-B (HT)	LacbiABCB4.1	-2.63	0	18225	6928
	707098	ABC-B (HT)	LacbiABCB5.1	-2.60	0	18954	7285
	297457	ABC-C	LacbiABCC2.1	-2.58	0	14297	5543
	301178	ABC-C	LacbiABCC3.1	-2.43	0.01	6912	2841
	307663	ABC-C	LacbiABCC3.2a	-2.69	0	8401	3121
	706294	ABC-C	LacbiABCC3.2b	-2.47	0.04	2312	937
	305568	ABC-C	LacbiABCC6.1	-2.21	0	11719	5311
Oidiodendron maius	193013	ABC-C	OidmaABCC5.1d	-2.23	5.17E-06	38.1	17.1
	104920	ABC-G	OidmaABCG1.1c	-3.38	0.004	59.8	17.7
	106738	ABC-G	OidmaABCG1.1e	-506.52	0	130.2	0.3
	103241	ABC-G	OidmaABCG3.1	-2.41	1.79E-05	30.3	12.6
Sebacina vermispora	68953	ABC-G	SebveABCG7.2	-2.68	0	180.5	67.4
Tulasnella calospora	71373	ABC-A	TulcaABCA1.1	-2.08	6.16E-04	28.7	13.8
	22914	ABC-C	TulcaABCC3.2a	-2.14	0.014	15.5	7.2
	32244	ABC-C	TulcaABCC4.1	-2.20	3.21E-06	43.5	19.8
	79164	ABC-D	TulcaABCD1.1	-2.40	4.59E-11	69.2	28.9
	234265	ABC-D	TulcaABCD2.1	-2.88	4.63E-18	87.1	30.3
	15356	ABC-F	TulcaABCF3.1	-3.60	2.30E-11	131.0	36.3
Suillus luteus	628180	ABC-B (FL)	SuiluABCB1.1	-2.43	3.02E-05	51.7	21.3
	809813	ABC-B (HT)	SuiluABCB4.1	-2.60	8.45E-25	239.6	92.1
	799345	ABC-C	SuiluABCC7.2	-2.23	1.23E-05	66.9	30.0
	807343	ABC-G	SuiluABCG2.1c	-7.33	2.58E-60	219.9	30.0
	807344	ABC-G	SuiluABCG2.1d	-2.22	3.29E-11	144.4	65.1
	442329	Ydr061w	SuiluABCI2.1	-4.14	3.95E-07	35.6	8.6
Piloderma croceum	820710	ABC-A	PilcrABCA1.1a	-2.08	9.23E-07	44.7	21.5
	822387	ABC-B (FL)	PilcrABCB1.1	-2.84	0	259.8	91.5
	60709	ABC-C	PilcrABCC3.2b	-2.16	1.49E-10	67.0	31.0
	801286	ABC-C	PilcrABCC7.1	-3.80	0	141.2	37.1
	816440	ABC-G	PilcrABCG1.1	-7.40	2.12E-08	17.9	2.4
	816614	ABC-G	PilcrABCG2.1b	-3.65	0	127.9	35.1
	816747	ABC-G	PilcrABCG3.1a	-3.07	1.97E-09	36.4	11.9
	813749	ABC-G	PilcrABCG4.1	-4.20	0	102.4	24.4
Hebeloma cylindrosporum	441821	ABC-B (HT)	HebcyABCB4.1	-2.00	0.039	33.9	17.0
	440458	ABC-E	HebcyABCE1.1	-2.58	1.49E-04	51.3	19.9
	447601	ABC-F	HebcyABCF1.1	-7.14	0.002	118.8	16.6
	439681	ABC-F	HebcyABCF2.1	-2.03	0.046	27.3	13.4
	63066	ABC-G	HebcyABCG4.1	-2.33	0.032	13.9	6.0
	441280	ABC-I	HebcyABCI1.1	-2.60	0.002	22.2	8.6

We were interested in how the fungal ABC genes, and, in particular, ABC transporters were influenced by the adaptations of fungi to their symbiotic lifestyle. These adaptations have evolved independently in different lineages of mycorrhiza-forming fungi [12], and they show a remarkable degree of evolutionary convergence. If ABC transporters do play a role in the process of mycorrhiza formation, their recruitment also occurred independently, and we might expect to find different transporters in different lineages depending on the type of mycorrhizal symbiosis. We would also expect to see the lineage-specific differences when comparing ABC transporter repertoire between mycorrhiza-forming fungi and their non-symbiotic relatives. Therefore, when possible, we have compared the sets of ABC genes found in the genomes of mycorrhiza-forming fungi, with the situation in related saprotrophic or plant-pathogenic species, paying attention to both quantitative and qualitative differences in the composition of ABC genes.

We have carried out a comprehensive analysis of ABC protein-encoding genes in the 25 fungal genomes that were sequenced by the MGI [12]. We have identified all predicted ABC genes and gene fragments, manually curated and annotated the gene models and classified them into the subfamilies according to the HUGO-proposed scheme [13]. The identified genes were further subdivided into smaller groups defined in our previous analyses [14, 15]. We have also analysed the data on expression of ABC transporter-encoding genes provided by members of the MGI consortium in order to identify those genes that might play a role in the establishment of mycorrhizal symbiosis. We could not identify any group of ABC transporters that would be unique for mycorrhiza-forming fungi. However, functions of many of identified genes are unknown, and it is possible that some of them play a certain role in the establishment of mycorrhizal interactions. Our data provide an insight into the diversity of ABC proteins in the newly sequenced genomes of mycorrhiza-forming fungi. Currently, it is not clear whether any fungal ABC transporters are implicated in the mycorrhiza formation. Results of our surveyshould provide a support for further studies on the possible role of fungal ABC transporters in the establishment of mycorrhizal symbiosis. Additionally, they will contribute to the better understanding of the evolution and dynamics of this protein family in the fungal kingdom.

## Results

**Fig. 1:**
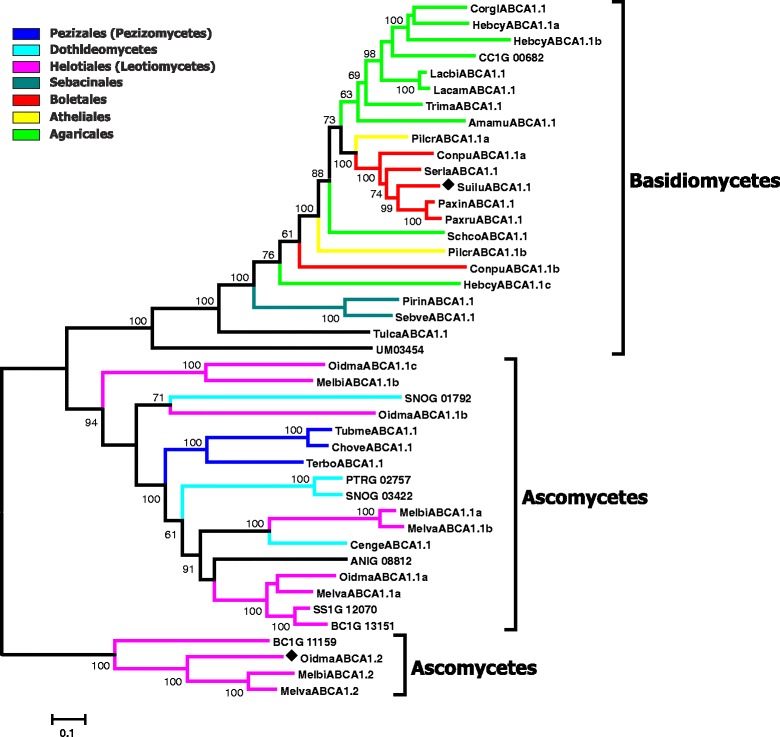
Maximum-likelihood phylogenetic tree of ABC-A transporters. Numbers next to the branching points indicate the relative support from 100 bootstrap replicates (only scores above 60 are shown). Ascomycetes- and basidiomycetes-specific branches are indicated. Selected fungal orders are indicated by colour code. The fungal species are indicated with the following abbreviations: RO3G, *Rhizopus oryzae*; Gloin, *Rhizomyces irregularis*; ANIG, *Aspergillus nidulans*; SNOG, *Phaeosphaeria* (*Stagonospora*) *nodorum*; PTRG, *Pyrenophora tritici-repentis*; Cenge, *Cenococcum geophilum*; SS1G, *Sclerotinia sclerotiorum*; BC1G, *Botryotinia fuckeliana* (*Botrytis cinerea*); Melbi, *Meliniomyces bicolor*; Melva, *M. variabilis*; Oidma, *Oidiodendron maius*; Tubme, *Tuber melanosporum*; Chove, *Choiromyces venosus*; Terbo, *Terfezia boudieri*; CC1G, *Coprinopsis cinerea*; Amamu, *Amanita muscaria*; Corgl, *Cortinarius glaucopus*; Hebcy, *Hebeloma cylindrosporum*; Lacam, *Laccaria amethystina*; Lacbi, *L. bicolor*; Schco, *Schizophyllum commune*; Trima, *Tricholoma matsutake*; Serla, *Serpula lacrymans*; Conpu, *Coniophora puteana*; Boled, *Boletus edulis*; Paxin, *Paxillus involutus*; Paxru, *P. rubicundus*; Pismi, *Pisolithus microcarpus*; Pisti, *P. tinctorius*; Sclci, *Scleroderma citrinum*; Suilu, *Suillus luteus*; Pilcr, *Piloderma croceum*; Tulca, *Tulasnella calospora*; Pirin, *Piriformospora indica*; Sebve, *Sebacina vermispora*; UM, *Ustilago maydis*. Names of *Saccharomyces cerevisiae* genes are listed without additional indices. Filled diamonds next to the sequence names indicate genes up-regulated in mycorrhiza-forming mycelium according to the transcriptomics data

**Fig. 2:**
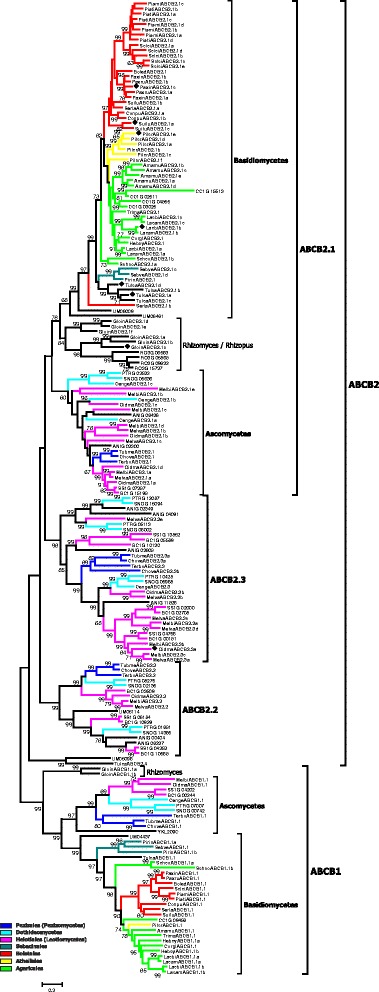
Maximum-likelihood phylogenetic tree of full-length ABC-B transporters. Numbers next to the branching points indicate the relative support from 100 bootstrap replicates (only scores above 60 are shown). The groups ABCB1 and ABCB2, the major clusters within them and ascomycetes-, basidiomycetes- and *Rhizomyces/Rhizopus*-specific branches are indicated. Selected fungal orders are indicated by colour code. Please refer to the legend to the Fig. 1 for the list of abbreviations of fungal names. Names of *S. cerevisiae* genes are listed without additional indices. Filled diamonds next to the sequence names indicate genes up-regulated in mycorrhiza-forming mycelium according to the transcriptomics data

**Fig. 3:**
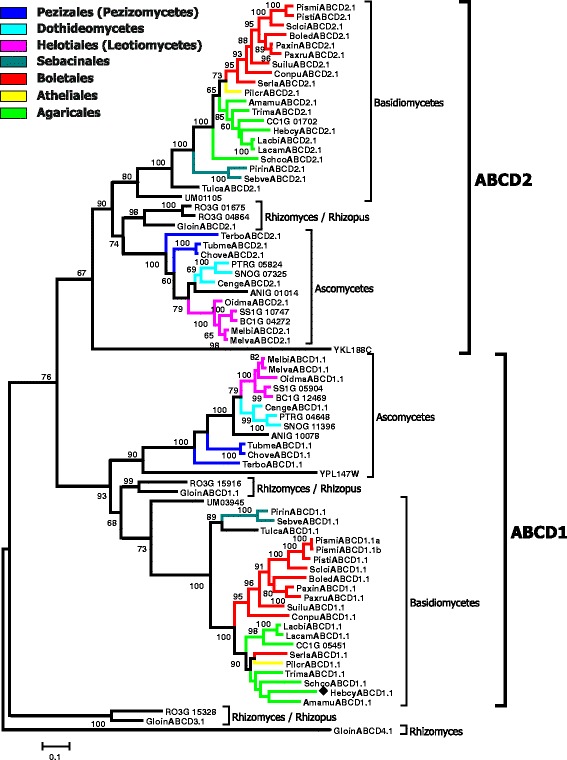
Maximum-likelihood phylogenetic tree of ABC-D transporters. Numbers next to the branching points indicate the relative support from 100 bootstrap replicates (only scores above 60 are shown). The groups ABCD1 and ABCD2 and ascomycetes-, basidiomycetes- and *Rhizomyces/Rhizopus*-specific branches are indicated. Selected fungal orders are indicated by colour code. Please refer to the legend to the Fig. 1 for the list of abbreviations of fungal names. Names of *S. cerevisiae* genes are listed without additional indices. Filled diamonds next to the sequence names indicate genes up-regulated in mycorrhiza-forming mycelium according to the transcriptomics data

**Fig. 4:**
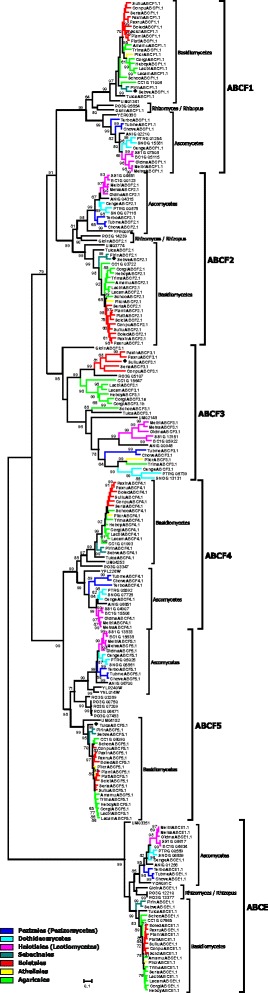
Maximum-likelihood phylogenetic tree of ABC-E and ABC-F proteins. Numbers next to the branching points indicate the relative support from 100 bootstrap replicates (only scores above 60 are shown). The groups ABC-E, ABCF1, ABCF2, ABCF3, ABCF4 and ABCF5 and ascomycetes-, basidiomycetes- and *Rhizomyces* / *Rhizopus*-specific branches are indicated. Selected fungal orders are indicated by colour code. Please refer to the legend to the Fig. 1 for the list of abbreviations of fungal names. Names of *S. cerevisiae* genes are listed without additional indices. Filled diamonds next to the sequence names indicate genes up-regulated in mycorrhiza-forming mycelium according to the transcriptomics data

**Fig. 5:**
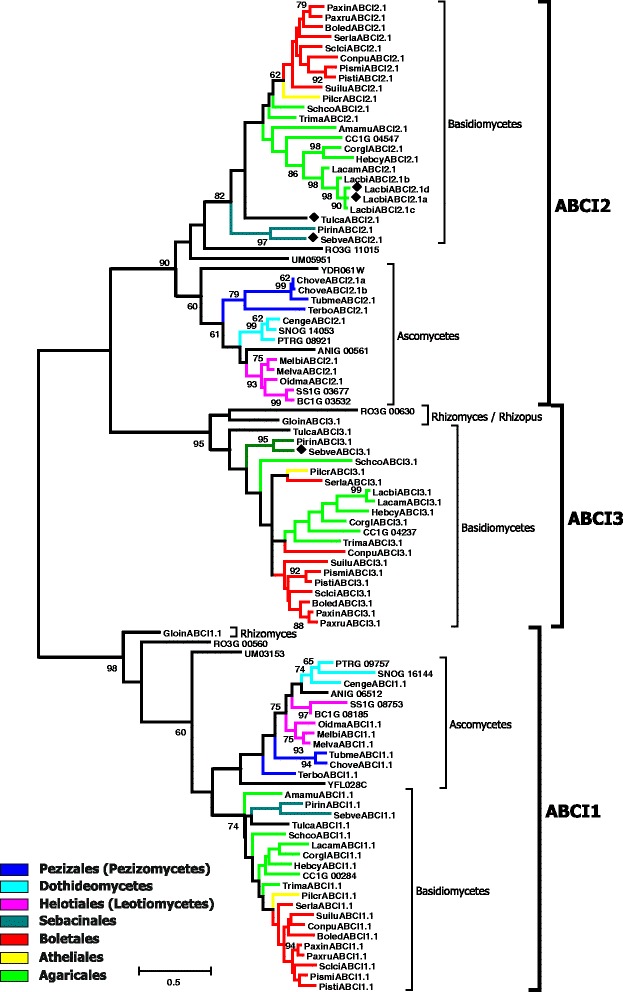
Maximum-likelihood phylogenetic tree of half-size ABC-I proteins. Numbers next to the branching points indicate the relative support from 100 bootstrap replicates (only scores above 60 are shown). The groups ABCI1, ABCI2 and ABCI3 and ascomycetes- and basidiomycetes -specific branches are indicated. Selected fungal orders are indicated by colour code. Please refer to the legend to the Fig. 1 for the list of abbreviations of fungal names. Names of *S. cerevisiae* genes are listed without additional indices. Filled diamonds next to the sequence names indicate genes up-regulated in mycorrhiza-forming mycelium according to the transcriptomics data

In the course of our inventory of ABC protein-encoding genes in the genomes of mycorrhiza-forming fungi, we have identified and manually curated and annotated more than 1300 gene models from 25 fungal species (Table 2 and Additional file 1; Additional file 2). 1022 of the analysed gene models are predicted to encode functional proteins. The manual curation of the identified genes has shown that 289 automatically generated gene models can be further improved (Additional file 2). The most common problems associated with the existing gene models were the positions of exon-intron boundaries and start and stop codons, but we have also encountered other kinds of errors, e.g. missing exons or cases when a single gene has been split into several (two or even three) models. The number of ABC genes in the analysed genomes varies to a great extent, from 29 in *Terfezia boudieri* to 72 in *Oidiodendron majus* (Table 2). Subsequently, we have reconstructed the evolutionary relationships of the identified ABC proteins (Figs. 1, 2, 3, 4, 5; Additional files 3, 4 and 5). Results of the phylogenetic analysis were in good agreement with our previous data [14, 15], as we could identify the same principal groups of fungal ABC proteins. In most trees ABC proteins from Basidiomycota and Ascomycota, respectively, were placed in separate and well supported monophyletic clades. A noticeable exception is the situation within the subfamily ABC-G, were the phylogenetic pattern is less clear (Additional file 5). The relationship between the groups ABCG1, ABCG2, ABCG3, ABCG4, and ABCG5 is not well resolved, and their positions on the tree vary depending on the species sampled for the analysis. In addition, there are some small clades that cannot be assigned to any of the larger groups. One of the reasons for that could be the presence of numerous paralogous genes in many species of ascomycetes. Additional efforts (i.e., broader sampling and inclusion of additional species from basal lineages of Asomycota and Basidiomycota) will be required to produce a robust phylogenetic reconstruction for this group.

Below we will present and discuss the most important findings of our analysis separately for each taxonomic group.

### *Phylum Glomeromycota*

Phylum Glomeromycota is one of the oldest fungal lineages that is highly unusual in many ways. Members of this group are obligate mutualistic symbionts forming a particular type of mycorrhizal interaction, known as arbuscular mycorrhiza (AM) [16–18]. We have identified 34 genes and 19 pseudogenes or gene fragments in the genome assembly of *Rhizophagus irregularis*, a species of Glomeromycota sequenced by the MGI consortium [18]. The distribution of the identified genes among the recognised subfamilies is shown in Table 2 and Additional file 2. Whereas the total number of ABC genes in *Rh. irregularis* is comparable with the numbers in other species included in our analysis, we could not identify representatives of some of the groups that are commonly found in the genomes of asco- and basidiomycetes (Additional file 1). Absence of genes homologous to *Saccharomyces cerevisiae MDL1*, *YEF3* and *YDR061W* is particularly remarkable as those groups are nearly ubiquitously present in the genomes of asco- and basidiomycetes analysed so far [14, 15], and *YEF3* (encoding the translation elongation factor 3) has shown to be essential for vegetative growth in *S. cerevisiae* [19]. We cannot exclude the possibility that the mentioned genes are present in the genome, but for some reason they are missing from the available genome assembly. Alternatively, some of them may have been lost by *Rh. irregularis* as a result of the adaptation to its obligate symbiotic lifestyle.

Some aspects of the organisation of ABC genes in *Rh. irregularis* show remarkable similarity with the zygomycete *Rhizopus oryzae* (Additional file 1) [14]*.* The similarity in the genome organization of *Rh. irregularis* and species of Mucoromycotina has been noticed previously [18]. One of the most prominent common features of *Rhizophagus* and *Rhizopus* is a presence of four ABC-D genes. Nearly all asco- and basidiomycetes have only two ABC-D genes. The products of those genes act as a heterodimeric peroxisomal long-chain fatty acid transporter. Remarkably, some of the species representing the ancient evolutionary lineages within fungal kingdom (e.g., *Rhizopus*, *Rhizophagus* and chytridiomycete *Spizellomyces punctatus* [14]) harbour additional genes of the subfamily ABC-D that in phylogenetic analysis are placed separately from the proteins of asco- and basidiomycetes (Fig. 3). The origin and function of those additional genes remains enigmatic.

Little is known about the role of *Rhizophagus* ABC transporters in the formation of arbuscular mycorrhiza. However, the transcript profiling has shown that one of the *Rh. irregularis* ABC-B transporters is arbuscle-specific, i.e. it is highly expressed in intraradical mycelium, but not during other stages of the *Rhizophagus* life cycle [17].

The unusual genetic organisation has substantially hampered the genome assembly of *Rh. irregularis* [16], and the available assembly is highly fragmented [18]. Therefore, we cannot exclude that some of the identified genes represent the different alleles of the same gene, and the actual number of ABC genes might be somewhat lower. At the same time, the detected gene fragments might be extended to full-length genes with further improvement of the genome assembly. It is also possible that some of the genes are still missing from the assembly (in particular, we would expect the presence of the *ScYEF3* homologue in *Rh. irregularis* genome).

### *Phylum Ascomycota*

#### *Class Leotiomycetes/*order* Helotiales*

**Fig. 6:**
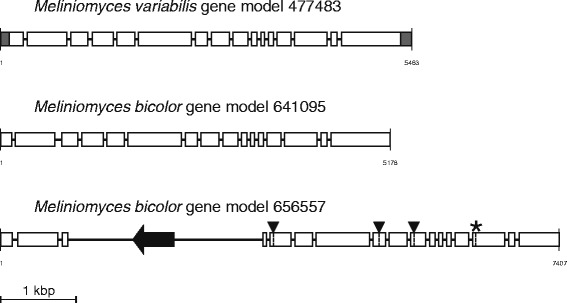
Inactivation of an ABC transporter-encoding gene due to insertion of a transposable element. The scheme illustrates three closely related homologous genes from the genomes of *M. bicolor* and *M. variabilis*. All three genes belong to the group ABCC5 of ABC-C transporters. The gene models Melva_477483 and Melbi_641095 are presumably intact, whereas the gene Melbi_656557 harbours a DNA transposon insertion that likely has caused its inactivation. All three gene models share the same exon-intron structure with 16 exons shown as open rectangles. 5´- and 3´-untranslated regions in the model Melva_477483 are shadowed grey. The exon 3 in the model Melbi_656557 is disrupted by the insertion of repetitive DNA carrying a putative transposase-encoding ORF (shown by a filled arrow). In comparison with the models Melva_477483 and Melbi_641095, the model Melbi_656557 also has three stop codons within the reading frame (indicated by filled triangles) and a putative translational frame-shift (indicated by an asterisk). Note that the last exon in the model Melbi_656557 is truncated. Accumulation of these mutations probably occurred after the gene has been inactivated by the transposon insertion

Three species from the order Helotiales were sequenced within the MGI project, *Oidiodendron maius*, *Meliniomyces bicolor* and *M. variabilis*. All three species form ericoid mycorrhiza with shrubs in the *Ericaceae* family (e.g., *Vaccinium* spp. and *Calluna vulgaris*). Both *Meliniomyces* spp. can also colonise roots of forest trees (pine, spruce, birch etc.) forming ectomycorrhiza-like symbiosis. The three species have the highest number of ABC genes among the species included in our analysis (Table 2 and Additional file 1). Interestingly, the number of ABC genes in these species negatively correlates with the genome size. *O. maius* has the highest number of ABC genes (72 genes), but the smallest genome assembly size, whereas *M. bicolor* has the lowest number of ABC genes among the three species, but the largest genome (Additional file 1). At the same time, *M. bicolor* has the highest number of pseudogenes, and we have observed at least six cases where *M. bicolor* ABC genes contained insertions of repetitive DNA (Fig. 6; Additional file 2). Extensive proliferation of repetitive DNA could be one of the factors contributing to the increase of genome size in *M. bicolor.*

**Fig. 7:**
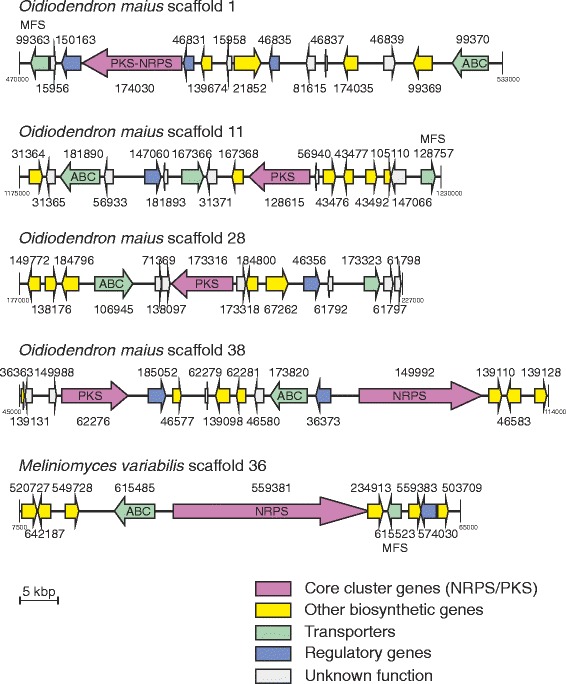
Association of fungal ABCC5 transporters with the secondary metabolism gene clusters. The scheme shows four scaffold fragments of *O. maius* and a single fragment from *M. variabilis*. All five fragments are predicted to carry clusters of genes involved in the biosynthesis of secondary metabolites (NRPS, PKS or hybrid PKS/NRPS clusters). The genes within the clusters are colour-coded according to their predicted function. All gene model numbers are indicated. In all cases, genes encoding predicted ABC transporters are located in the immediate vicinity of the core gene of the cluster

A peculiar feature of all three species is a high number of genes belonging to the group ABCC5 of ABC-C transporters. Members of this group are often found associated with the secondary metabolism clusters. Indeed, we have observed that 4 *O. maius* genes and one *M. variabilis* gene were located in the immediate vicinity of polyketide synthase (PKS), nonribosomal peptide synthetase (NRPS) or hybrid NRPS/PKS clusters (Fig. 7). At the same time, genes of this group were the ones most often targeted by insertions of repetitive DNA, apparently resulting in their pseudogenisation. We have detected this kind of insertions in three *M. bicolor* genes and in one *M. variabilis* gene.

The number of ABC genes in the three mycorrhiza-forming species of the order Helotiales is considerably higher than in two phytopathogenic species from the same order, *Sclerotinia sclerotiorum* and *Botryotinia fuckeliana* (anamorph *Botrytis cinerea*) [14] (Additional file 1). The higher number of genes representing the group ABCC5 is probably linked with the higher number of secondary metabolism gene clusters in those species. Representatives of all four groups over-represented in mycorrhiza-forming Helotiales were identified among the genes with the increased transcript abundance in mycorrhiza-forming mycelium (fold change > 2, FDR < 0.05) (Table 3; Additional file 6) [12]. This observation indicates that at least some of them might have a role in the establishment of the mycorrhiza symbiosis.

### *Class Dothideomycetes*

*Cenococcum geophilum* is the only known ectomycorrhizal species within the class Dothideomycetes. We have detected 35 genes encoding predicted ABC protein together with 5 putative pseudogenes or gene fragments in the genome of *C. geophilum* (Table 2 and Additional file 1; Additional file 2). Contrary to the situation observed within the order Helotiales, the number of ABC genes in *C. geophilum* is somewhat lower than the corresponding numbers in the genomes of the phytopathogenic dothideomycetes *Pyrenophora tritici-repentis* and *Stagonospora nodorum* [14]. Those differences are mainly due to the lower number of full-length ABC-B and ABC-G transporters in *C. geophilum*. Nevertheless, the sets of ABC genes in the genomes of all three representatives of Dothideomycetes show high degree of similarity and there are only a few noteworthy differences (Additional file 1).

### *Class Pezizomycetes/* order* Pezizales*

The order Pezizales is represented in our analysis by three species, *Tuber melanosporum* (family *Tuberaceae*), *Choiromyces venosus* (*Tuberaceae*) and *Terfezia boudieri* (*Pezizaceae*). All three species are characterised by rather low number of ABC protein-encoding genes (Table 2 and Additional file 1; Additional file 2), and *T. boudieri* has the lowest number of ABC genes (29 genes) among mycorrhiza-forming species included in our analysis and, as far as known, among all analysed filamentous ascomycetes [14]. In particular, numbers of ABC-G, ABC-C and full-length ABC-B transporters in the analysed species of Pezizales are lower than in other filamentous ascomycetes (Additional file 1).

The existence of 27 ABC transporters in the genome of *T. melanosporum* has been reported previously [20]. After careful examination and manual curation of the available gene models, we have identified five cases of misannotation, when a single ABC transporter-encoding gene was split into two separate non-overlapping models (Additional file 2). After their correction, we concluded that the genome of *T. melanosporum* has 24 genes for ABC transporters and 8 genes encoding ABC proteins without transmembrane domains.

The sets of ABC proteins in all three analysed species of Pezizales show a high level of similarity, and in most cases the corresponding homologues form well-supported groups in our phylogenetic analysis. The presence of the genes representing the group ABCC4 in all three species of Pezizales is noteworthy, as this type of transporters is quite uncommon in ascomycetes.

The transcript abundance of one of the *T. melanosporum* ABC-G transporters was increased in the ectomycorrhizal root tips compared to the free-living mycelium [20], indicating its potential role in the establishment of the mycorrhizal symbiosis.

### *Phylum Basidiomycota/*class* Agaricomycetes*

#### *Order Agaricales*

We have analysed genome sequences of six mycorrhiza-forming species of Agaricales: *Amanita muscaria*, *Cortinarius glaucopus*, *Hebeloma cylindrosporum*, *Laccaria amethystina*, *L. bicolor* and *Tricholoma matsutake*. The data on *L. bicolor* ABC genes were taken from our previous analysis [15]. All six species are engaged in ectomycorrhiza symbiosis with a wide range of forest trees, including both conifers and broad-leaved trees. The numbers of identified ABC genes in the genomes of mycorrhiza-forming Agaricales range from 33 genes in *A. muscaria* to 47 genes in *C. glaucopus*. All of the analysed species, except for *H. cylindrosporum*, are characterised by the presence of high numbers of gene fragments and putative pseudogenes related to the functional ABC protein-encoding genes, ranging from 15 in *A. muscaria* to 53 in *L. amethystina* (Additional file 1).

There is a considerable similarity between the sets of ABC genes in the genomes of the analysed mycorrhiza-forming species and of saprotrophic representatives of Agaricales, *Coprinopsis cinerea* [14] and *Agaricus bisporus* (our unpublished data), with the differences being mostly quantitative. However, both saprotrophic species have a considerably lower number of putative pseudogenes or gene fragments in their genomes. The set of ABC genes in *A. muscaria* is quite distinct, as its genome lacks some of the genes present in the remaining species (Additional file 1).

The distinctive features of *C. glaucopus* genome are the high number of the genes belonging to the groups ABCC4 and ABCC7, and the absence of ABC-D transporters and of the group ABCC6. The group ABCC4 is made up by proteins of unknown function nearly ubiquitously present in Agaricomycotina. There are 11 genes of this group in the genome of *C. glaucopus*, whereas their number in other analysed species of Agaricales ranges from 1 to 5. The group has undergone a remarkable amplification in Polyporales [15] with some species having up to 16 genes.

A common feature of the analysed species of Agaricales (including saprotrophs) is the high number of genes belonging to the group ABCB6 of half-size ABC-B transporters. To our current knowledge, the group is found only in the species of Agaricomycotina [15], and the biological role of the corresponding proteins remains obscure.

Analyses of the gene expression during mycorrhiza formation have been performed in two species of Agaricales, *L. bicolor* and *H. cylindrosporum* (Tables 3 and 4; Additional file 6) [12]. The identification of a group ABCB6 half-size ABC-B transporter and ScYDR061W homologues in *L. bicolor* experiment is noteworthy, as biological functions of those two groups have not been elucidated in any of the fungal species yet.

### *Order Boletales*

We have analysed the genomes of seven mycorrhiza-forming species of Boletales representing four distinct evolutionary lineages within the order [21]: Suillineae (*Suillus luteus*), Sclerodermatineae (*Scleroderma citrinum*, *Pisolithus microcarpus* and *P. tinctorius*), Paxillineae (*Paxillus involutus* and *P. rubicundus*), and Boletinae (*Boletus edulis*). We have also compared the data from these 7 species with the sets of ABC transporters identified in two wood-degrading species of Boletales (*Coniophora puteana* and *Serpula lacrymans* [15]) representing the basal lineages within the order [21]. The numbers of ABC-protein encoding genes in the analysed mycorrhiza-forming species of Boletales range from 30 in *B. edulis* to 42 in *P. tinctorius* (Table 2 and Additional file 1; Additional file 2). Genomes of many species (in particular, *P. involutus* and *P. microcarpus*) also harbour a high number of gene fragments and putative pseudogenes derived from ABC transporter-encoding genes.

There are no pronounced differences in sets of ABC proteins between wood-degrading and mycorrhiza-forming species of Boletales. However, some of the ABC transporters identified in Boletales have noteworthy features. One of the interesting finding of our analysis was the observation that the predicted proteins of the group ABCB4 of half-size ABC-B transporters in *B. edulis*, *Paxillus* spp., *Pisolithus* spp. and *S. citrinum* differ considerably from their counterparts in other species of Agaricomycotina, including the remaining members of Boletales (*S. luteus*, *C. puteana* and *S. lacrymans*). Sequences from the six mentioned species were also clearly separated in the phylogenetic analysis, forming an isolated branch on the phylogenetic tree (Additional file 3). Remarkably, the sequences of their predicted transmembrane domains had a higher level of sequence similarity to the ABCB4 proteins from other fungi than the sequences of their nucleotide-binding domains. Most plausible explanation for this observation would be that the changes in the nucleotide sequence ABCB4 gene occurred in the common ancestor of Boletineae, Paxillineae and Sclerodermatineae after the separation of the lineage of Suillineae. It is not entirely clear what could be the cause of these changes, and whether the derived genes retained their biological function. The assignment of these proteins to the group ABCB4 is tentative, and further analysis will be required to clarify their origin and phylogenetic affinities.

Another distinct feature of Boletales is a presence of the gene encoding an unusual ABC-C transporter with a predicted AMP-binding N-terminal domain. This gene was identified before in *S. lacrymans*, *C. puteana* and polyporoid fungus *Ceriporiopsis subvermispora* [15]. We could detect this gene in all the analysed species of Boletales except for *S. luteus*. The identified sequences were placed in a separate isolated group in our phylogenetic analysis. Remarkably, in all species the sequence coding for the predicted AMP-binding domain is present, further strengthening our suggestion that this domain indeed makes part of the transporter protein. It has been hypothesised that this unusual structure could be essential for tight coupling of the biosynthesis of yet unknown metabolite with its transport, and indeed, the gene is associated with an uncharacterised NRPS clusters in *C. puteana* and *S. lacrymans* [15]. However, we could not detect similar associations of this gene with secondary metabolism gene clusters in mycorrhiza-forming species of Boletales.

Similarly to Agaricales, species of Boletales have a diverse set of genes of unknown function belonging to the Agaricomycotina-specific group ABCB6 of half-size ABC-B transporters. Transcriptomics experiments have identified several ABC genes of Boletales up-regulated in ectomycorrhiza-forming mycelium (Table 3; Additional file 6) [12].

### *Order Atheliales*

The small order Atheliales is represented in our analysis by a single species, a broad host-range mycorrhizal symbiont *Piloderma croceum*, widespread in boreal and temperate forests. Atheliales are most closely related to Boletales and Agaricales [22]. However, the number of ABC genes in *Piloderma* (48) exceeds the numbers found in the analysed species of those two orders. It also has a considerable number (15) of gene fragments and putative pseudogenes. The set of ABC transporters found in the genome of *P. croceum* is characterised in particular by the high diversity of ABC-G transporters, with some of the genes without counterparts in other analysed species of Agaricomycotina (Additional file 5). Other noteworthy features of *P. croceum* are a high number of full-length ABC-B transporters and a diverse set of half-size ABCB6 genes. Three *P. croceum* ABC genes were significantly induced during mycorrhiza formation: full-length ABC-B (ABCB2) transporter, half-size ABC-B transporter (ABCB6), and a half-size ABC-G transporter (ABCG6) (Table 3; Additional file 6) [12].

### *Order Cantharellales*

This order is one of the ancient lineages within Agaricomycotina [22]. It is represented in our analysis by *Tulasnella calospora*, a widespread symbiont of green orchids with the world-wide distribution. In association with its host plants, *T. calospora* forms a distinct type of endomycorrhiza known as orchid-type mycorrhiza. The genome of *T. calospora* harbours 40 predicted ABC genes and 3 gene fragments likely derived from the intact ABC genes. The identified set of ABC genes is in many aspects “ordinary”, i.e. there are no prominent distinctive features. All of the groups of ABC proteins commonly found in Agaricomycotina are represented, and there are no “odd” genes. We could only mention here the relatively high number of full-length ABC-B genes, and the lower diversity of the group ABCB6 of half-size ABC-B transporters as compared with the species of Agaricales and Boletales.

Transcriptomics analysis has shown that 9 genes encoding ABC transporters and 2 genes encoding soluble ABC proteins were up-regulated in *T. calospora* during mycorrhiza formation (Table 3; Additional file 6) [12].

### *Order Sebacinales*

Together with the order Cantharellales, this is one of the basal lineages within Agaricomycotina. In our analysis, we have included the orchid symbiont *Sebacina vermifera*, which was compared with the root endophyte *Piriformospora indica* [23]. The sets of ABC genes in the analysed species show considerable similarity (Additional file 1). The sequences of the half-size ABC-B transporters from the two species, which we provisionally assign to group ABCB4, are highly unusual and differ significantly from the corresponding sequences found in other Agaricomycotina, in particular in the amino acid sequence of their nucleotide-binding domain. Both proteins are also considerably shorter than ABCB4 proteins in other species of fungi. In our phylogenetic analysis the two sequences formed an isolated group of unclear relationship (Additional file 4). Their affinities with the group ABCB4 require additional studies.

Transcriptomics analysis has identified a number of *S. vermifera* ABC genes as being significantly up-regulated in mycorrhiza-forming mycelium (Table 3).

## Discussion

Studies on several model plant species have demonstrated that particular plant ABC transporters are required for the formation of the arbuscular mycorrhiza [8–10]. At the same time, the extensive exchange of nutrients between the both partners engaged in mycorrhiza formation probably requires the action of additional transporter proteins, not only on the host side, but also on the side of the colonising fungus. Currently, it remains unknown whether any of fungal ABC transporters contribute to the formation of any type of mycorrhizal symbiosis.

Our inventory provides an important insight into the diversity of ABC transporters and soluble ABC proteins in the genomes of mycorrhiza-forming fungi. However, the interpretation of the obtained results is considerably hampered by the scarcity of the available information on the biological functions of fungal ABC transporters. Results of our inventory indicate that there are no principal differences in sets of ABC proteins encoded by mycorrhiza-forming fungi, on one hand, and saprotrophic or phytopathogenic fungi, on other hand. We could not identify any ABC transporters that could be classified as “mycorrhiza-specific”. Moreover, the differences between members of various evolutionary lineages of fungi are much more pronounced than between the species occupying different ecological niches. Thus, the principal question, whether any of fungal ABC transporters play a role in the establishment and functioning of mycorrhizal symbiosis, remains open. However, the available data on gene expression in several fungal species indicate that some ABC transporter-encoding genes are transcriptionally up-regulated during the mycorrhiza formation [12, 17, 20, 24]. The performed transcriptomics studies have repeatedly identified members of some groups of ABC transporters as induced upon mycorrhiza formation, e.g. full-length ABC-B transporters of the group ABCB2, half-size ABC-B transporters of the group ABCB6, the transporters belonging to the group ABCG6 and the soluble ABC proteins of the group ABCI2. The group ABCB2 is widely distributed among fungi and nearly ubiquitously present in members of different lineages. Transporters of this group are also known as MDR transporters, as some of them contribute to the resistance to antifungal compounds. The formation of paralogous genes within this group is evident from our phylogenetic analysis (Fig. 2). It is tempting to speculate that some of those paralogous copies have acquired new functions as a result of an adaptation to the symbiotic life style.

The group ABCB6 is particularly interesting as it seems to be specific to the species of Agaricomycotina. No members of this group have been identified up till now from other fungal lineages. Members of this group have a typical structure of half-size ABC transporters, with N-terminal transmembrane domain and C-terminal nucleotide binding domain. They show a considerable degree of diversification within Agaricomycotina. In our phylogenetic analysis, four clusters with high bootstrap support could be recognised within the group. The highest diversity of its members is observed in the crown groups of Agaricomycotina, i.e. orders of Agaricales and Boletales, whereas the diversity is lower in basal lineages (like Sebacinales and Cantharellales). ABCB6 genes have been identified in all analysed Agaricomycotina regardless of their life style and ecological niche with the sole exception of opportunistic pathogen *Cryptococcus neoformans*. None of the members of this group has been characterised experimentally so far, but their wide distribution and a high diversity indicate that they might play an important role in basidiomycetes.

ABCG6 is another group encompassing transporters of unknown function. It is widely but unevenly distributed among fungal lineages, as it is completely missing, for example, from all analysed species of Boletales. The last group ABCI2 is made up by the homologues of the uncharacterised *S. cerevisiae* gene YDR061W. Members of this group are ubiquitously present in fungal genomes, but their biological role remains obscure.

Our analysis and the available transcriptomics data have pinpointed those of ABC transporters that might contribute to the mycorrhiza formation in different fungal lineages. Whereas some of the transporters have been repeatedly identified in several species, there is no uniform picture, and many of the genes have been up-regulated in just a single species. This is not particularly surprising, as mycorrhiza-forming fungi are very diverse phylogenetically, and the ability to form mycorrhiza-like symbiosis has apparently evolved independently in several evolutionary lineages of fungi [7]. Furthermore, there are considerable structural and physiological differences between various types of mycorrhiza (ectomycorrhiza, ericoid, orchid and arbuscular mycorrhiza), and fungi engaged in each of those type of symbiotic interactions likely have evolved their own specific adaptations. Additional work will be required to clarify the differences in nutrient flow and exchange and in signalling in various types of mycorrhiza symbiosis and, in particular, contribution of different classes of transporter proteins to those processes.

## Conclusions

We report here the results of our large-scale effort towards the complete inventory of ABC protein-encoding genes in the genomes of mycorrhiza-forming fungi. Results of our inventory show that the sets of ABC genes differ considerably in various phylogenetic lineages, both in total number of genes and in their distribution among the subfamilies. Genomes of Helotiales are particularly rich in ABC transporter-encoding genes, having almost twice as many corresponding genes as species of Pezizales or some Boletales. Therefore, it is difficult to draw a common pattern for all the analysed groups. The comparison between the mycorrhiza-forming species and their saprotrophic or parasitic relatives also revealed the considerable differences between various evolutionary lineages of fungi. Thus, mycorrhiza-forming species of Helotiales have higher numbers of ABC genes compared with the analysed plant pathogens of the same order, whereas in Dothideomycetes we have observed an opposite trend (however, here the compared species were not very closely related). In Agaricales and Boletales the differences between the saprotrophs and mycorrhiza-formers were even less pronounced. However, in many of the analysed species we have observed the formation of species- or lineage-specific groups of paralogous genes. Neofunctionalisation within such groups might be one of the forces contributing to the adaptation to the symbiotic life style.

Our results also contribute to the better knowledge of the evolution and diversity of fungal soluble ABC proteins and ABC transporters. We provide the first data on ABC protein-coding genes for the members of one fungal phylum (Glomeromycota) and four fungal orders (Pezizales, Atheliales, Cantharellales and Sebacinales). The information on ABC genes from the genome of the glomeromycete *Rhizomyces irregularis* is particularly important for the understanding of the evolution of this gene family in the ancient lineages of the fungal evolutionary tree before the divergence of lineages leading to modern Ascomycota and Basidiomycota.

Finally, the reported data should provide a contribution to prioritising target genes for the forthcoming functional characterisation, and in this way essentially advance the studies of fungal ABC transporters in general.

## Methods

### Genome sequencing

The genomic DNA sequences of the mycorrhiza-forming fungi (except for the previously sequenced species *Laccaria bicolor* [24], *Tuber melanosporum* [20] and *Piriformospora indica* [23]) were obtained at the Joint Genome Institute (JGI) of the US Department of Energy (DOE) in Walnut Creek (California), as part of the Mycorrhizal Genomic Initiative (MGI) project. The genomes were produced as described by [12]. The results from gene prediction and annotation in the above genomes are available for searching in Mycocosm [25] at the JGI portal (http://jgi.doe.gov/fungi).

### ABC genes identification

To identify gene loci encoding ABC proteins in the fungal genomes, multiple tblastn and blastp searches against selected genomes were performed at the website of the Fungal Genomics Program of the Department of Energy Joint Genome Institute (JGI) [25]. Sequences of *S. cerevisiae* and *Coprinopsis cinerea* ABC proteins representing all known subfamilies were used as queries. All hits producing E-values below 10^-4^ were further analysed. Gene models were manually curated and, when necessary, the positions of N and C termini and exon-intron boundaries were adjusted.

### Phylogenetic analysis

Phylogenetic analysis was performed with the program package MEGA6 [26]. Initial assignment of identified sequences to the subfamilies was done separately for every species in a following way. All proteins identified in a particular genome were aligned with known ABC proteins from *S. cerevisiae*, *Aspergillus nidulans*, *C. cinerea* and *Ustilago maydis* using MUSCLE algorithm integrated in MEGA6 package. Neighbor-joining phylogenetic trees were reconstituted based on the obtained alignments. All recognized subfamilies of ABC proteins could be separated on trees produced in such way, allowing the fast assignment of the analysed sequences to the particular subfamily. The detailed analysis was performed separately for each subfamily and, in the case of ABC-B transporters, also for full-length and half-size proteins. Multiple sequence alignments were constructed with the MUSCLE algorithm integrated in MEGA6 package using default settings. Alignments were quality trimmed with Gblocks 0.91 b [27]. Both full-length and trimmed alignments were used to produce phylogenetic trees. Neighbor-joining trees were constructed using Jones-Taylor-Thornton substitution model with 500 bootstrap replications. Maximum-likelihood trees were obtained using Jones-Taylor-Thornton model [28] with 100 bootstrap replications. Although the topologies of the phylogenetic trees produced with the different algorithms showed some minor differences, the same major groups of ABC proteins were recognized in all reconstructions.

### Availability of supporting data

The data sets supporting the results of this article are available in the TreeBase repository under the following link: http://purl.org/phylo/treebase/phylows/study/TB2:S17878.

## Additional files

Additional file 1: Table S1. Numbers of ABC protein-encoding genes in the analyzed species of fungi. (XLSX 16 kb) Additional file 2: Table S2. Loci and amino acid sequences of ABC proteins identified in this work. (XLS 1484 kb) Additional file 3: Figure S1. Maximum-likelihood phylogenetic tree of half-size ABC-B transporters. Numbers next to the branching points indicate the relative support from 100 bootstrap replicates (only scores above 60 are shown). The groups ABCB3, ABCB4, ABCB5 and ABCB6, the major clusters within them, the two outlying groups formed by the sequences from Boletales and Sebacinales, and ascomycetes-, basidiomycetes- and *Rhizomyces* / *Rhizopus*-specific branches are indicated. Selected fungal orders are indicated by colour code. Please refer to the legend to the Fig. 1 for the list of abbreviations of fungal names. Names of *S. cerevisiae* genes are listed without additional indices. Filled diamonds next to the sequence names indicate genes up-regulated in mycorrhiza-forming mycelium according to the transcriptomics data. (PDF 43 kb) Additional file 4: Figure S2. Maximum-likelihood phylogenetic tree of ABC-C transporters. Numbers next to the branching points indicate the relative support from 100 bootstrap replicates (only scores above 60 are shown). The groups ABCC1, ABCC2, ABCC3, ABCC4, ABCC5, ABCC6 and ABCC7, the outlying group ABCC7.5, and ascomycetes-, basidiomycetes- and *Rhizomyces / Rhizopus*-specific branches are indicated. Selected fungal orders are indicated by colour code. Please refer to the legend to the Fig. 1 for the list of abbreviations of fungal names. Names of *S. cerevisiae* genes are listed without additional indices. Filled diamonds next to the sequence names indicate genes up-regulated in mycorrhiza-forming mycelium according to the transcriptomics data. (PDF 1392 kb) Additional file 5: Figure S3. Maximum-likelihood phylogenetic tree of ABC-G transporters. Numbers next to the branching points indicate the relative support from 100 bootstrap replicates (only scores above 60 are shown). Groups ABCG1-5, ABCG6 and ABCG7, the major clusters within them and ascomycetes-, basidiomycetes- and *Rhizomyces / Rhizopus*-specific branches are indicated. Selected fungal orders are indicated by colour code. Please refer to the legend to the Fig. 1 for the list of abbreviations of fungal names. Names of *S. cerevisiae* genes are listed without additional indices. Filled diamonds next to the sequence names indicate genes up-regulated in mycorrhiza-forming mycelium according to the transcriptomics data. (PDF 1381 kb) Additional file 6: Table S3. Expression levels of ABC protein-encoding genes in free-living mycelium and in mycorrhiza-forming mycelium in the selected mycorrhiza-forming fungi. (XLSX 56 kb)
