# Supplementary figures and images for: Diversity and evolution of ABC proteins in mycorrhiza-forming fungi
Source: BMC Evol Biol. 2015 Dec 28;15:249. doi: 10.1186/s12862-015-0526-7 (PMC4692070; doi:10.1186/s12862-015-0526-7)

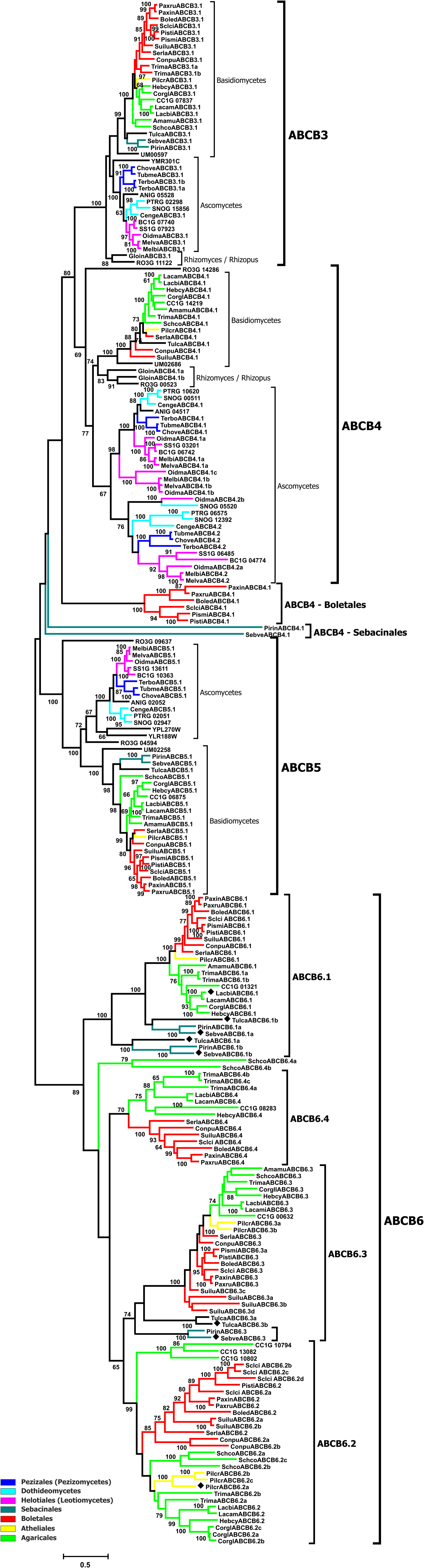

Supplement: Additional file 3: Figure S1. — Maximum-likelihood phylogenetic tree of half-size ABC-B transporters. Numbers next to the branching points indicate the relative support from 100 bootstrap replicates (only scores above 60 are shown). The groups ABCB3, ABCB4, ABCB5 and ABCB6, the major clusters within them, the two outlying groups formed by the sequences from Boletales and Sebacinales, and ascomycetes-, basidiomycetes- and Rhizomyces / Rhizopus-specific branches are indicated. Selected fungal orders are indicated by colour code. Please refer to the legend to the Fig. 1 for the list of abbreviations of fungal names. Names of S. cerevisiae genes are listed without additional indices. Filled diamonds next to the sequence names indicate genes up-regulated in mycorrhiza-forming mycelium according to the transcriptomics data. (PDF 43 kb) [file 12862_2015_526_MOESM3_ESM.pdf]

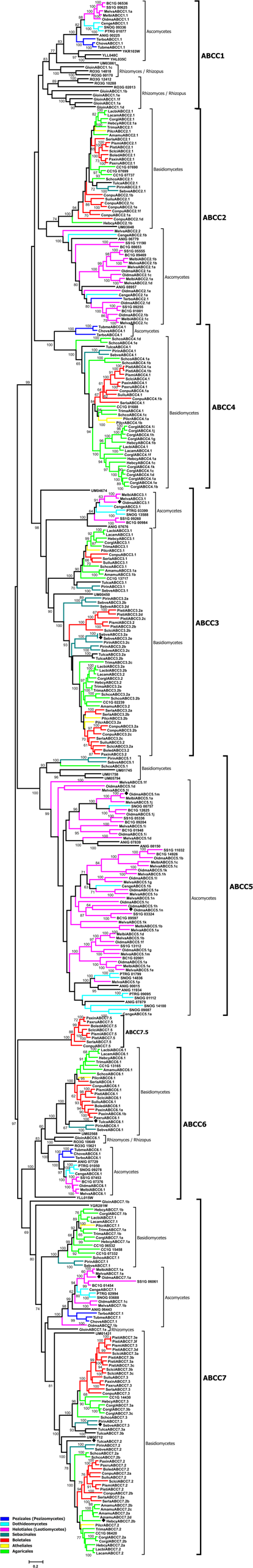

Supplement: Additional file 4: Figure S2. — Maximum-likelihood phylogenetic tree of ABC-C transporters. Numbers next to the branching points indicate the relative support from 100 bootstrap replicates (only scores above 60 are shown). The groups ABCC1, ABCC2, ABCC3, ABCC4, ABCC5, ABCC6 and ABCC7, the outlying group ABCC7.5, and ascomycetes-, basidiomycetes- and Rhizomyces / Rhizopus-specific branches are indicated. Selected fungal orders are indicated by colour code. Please refer to the legend to the Fig. 1 for the list of abbreviations of fungal names. Names of S. cerevisiae genes are listed without additional indices. Filled diamonds next to the sequence names indicate genes up-regulated in mycorrhiza-forming mycelium according to the transcriptomics data. (PDF 1392 kb) [file 12862_2015_526_MOESM4_ESM.pdf]

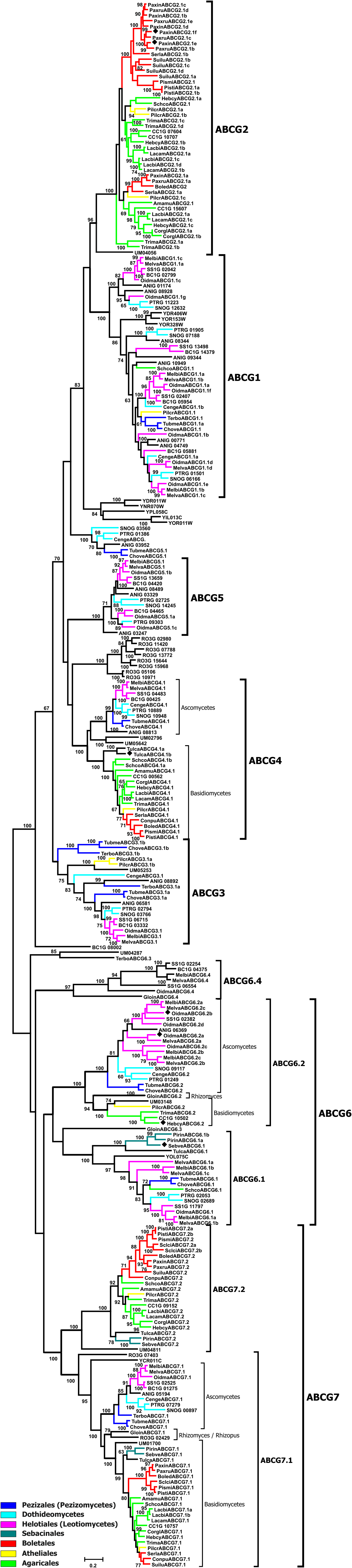

Supplement: Additional file 5: Figure S3. — Maximum-likelihood phylogenetic tree of ABC-G transporters. Numbers next to the branching points indicate the relative support from 100 bootstrap replicates (only scores above 60 are shown). Groups ABCG1-5, ABCG6 and ABCG7, the major clusters within them and ascomycetes-, basidiomycetes- and Rhizomyces / Rhizopus-specific branches are indicated. Selected fungal orders are indicated by colour code. Please refer to the legend to the Fig. 1 for the list of abbreviations of fungal names. Names of S. cerevisiae genes are listed without additional indices. Filled diamonds next to the sequence names indicate genes up-regulated in mycorrhiza-forming mycelium according to the transcriptomics data. (PDF 1381 kb) [file 12862_2015_526_MOESM5_ESM.pdf]
